# Gluteus Medius for Individuals with Chronic Ankle Instability: Assessing Muscle Activity

**DOI:** 10.5114/jhk/190267

**Published:** 2024-07-17

**Authors:** Lijiang Luan, Zhengliang Xia, Roger Adams, Charlotte Ganderton, Oren Tirosh, Doa El-Ansary, Adrian Pranata, Jia Han

**Affiliations:** 1 School of Exercise and Health, Shanghai University of Sport, Shanghai, China.; 2 Discipline of Physiotherapy, School of Health Sciences, University of Sydney, Sydney, Australia.; 3 Research Institute for Sport and Exercise, University of Canberra, Canberra, Australia.; 4 Department of Nursing and Allied Health, School of Health Sciences, Swinburne University of Technology, Melbourne, Australia.; 5 School of Health and Biomedical Sciences, Royal Melbourne Institute of Technology (RMIT University), Melbourne, Australia.; 6 College of Rehabilitation Sciences, Shanghai University of Medicine and Health Sciences, Shanghai, China.; 7 Department of Surgery, Melbourne Medical School, University of Melbourne, Melbourne, Australia.

**Keywords:** functional performance, physical activities, electromyography, systematic review

## Abstract

Emerging evidence has suggested that gluteus medius (GM) muscle activity may be critical for functional performance in individuals with chronic ankle instability (CAI). This study aimed to systematically review the literature to determine whether there are differences in GM muscle activity between individuals with and without CAI. A comprehensive search in PubMed, Embase, Cochrane Library, Web of Science, EBSCO, and PEDro databases was undertaken from the year of inception to 10 June 2024. Studies that investigated GM muscle activity during physical activities in healthy controls or copers and individuals with CAI were included. The quality assessment was conducted using the Newcastle-Ottawa Quality scale (NOS). After review, forty studies (1840 participants) were included; NOS scoring for the included studies ranged from 5/9 to 9/9 stars. GM activity was reported for seven activities: walking (14 studies), stance-transition (four studies), jump-landing (13 studies), perturbation (six studies), balance (four studies), cutting (three studies), and other functional exercises (seven studies). The outcome measures selected to examine each task varied across studies, and electromyography (EMG) results were inconsistent. Overall, although the quality of the available studies was generally high, there were substantial methodological differences, and the activity of GM muscles in CAI participants compared to controls was equivocal. A consensus on standardization of GM muscle activity assessment reporting should be established to guide future studies.

## Introduction

Ankle sprain is one of the most common musculoskeletal injuries among males and females involved in daily activities and sports (Labanca et al., 2021). Following an initial ankle sprain, a high proportion (40%) of individuals develop chronic ankle instability (CAI) (Delahunt et al., 2018; Doherty et al., 2016), a condition characterized by ongoing symptoms of pain, decreased ankle range of motion (ROM), perceptions of having unstable ankles or frequent episodes of the ankle giving way, that persists for more than a year, with reduced self-reported function (Han et al., 2015; Hertel and Corbett, 2019). There is growing evidence that those who suffer from CAI may not only have poor ankle joint performance, but also demonstrate deficits in the physical functioning of the whole lower limb (Labanca et al., 2021; Luan et al., 2021).

For improving functional performance of lower limbs in individuals with CAI, it is necessary to enhance the ability to contract and coordinate the hip muscles, since hip muscles play an important role in posture control and sport performance (Han et al., 2015; Labanca et al., 2021; Moisan et al., 2017; Wilczyński et al., 2022). Among the hip muscles, the gluteus medius (GM) muscle is critical for hip abduction. It plays a vital role in controlling lateral pelvic stability and femoral rotation in functional movements (DeJong et al., 2022), which can directly impact the kinetic function of the lower limb, particularly in a single leg stance (DeJong et al., 2019, 2020a).

Considering research on GM muscle in individuals with CAI, muscle activity is currently the main concern, given that this is a determinant of the generation of force needed to control the movements of the lower limb (DeJong et al., 2022; Koldenhoven et al., 2019a). GM activity is often measured using electromyography (EMG) that records the amplitude and timing of muscle activity during functional tasks (McCrary et al., 2018). This measurement has been reported to have high reliability and validity (Sadler et al., 2020). Moreover, previous studies have reported that individuals with CAI have changes in GM muscle activity, as measured by EMG, compared to healthy controls (Fatima et al., 2020; Moisan et al., 2017; Northeast et al., 2018). For example, the GM muscle in individuals with CAI could reflect an increase or a decrease in muscle activity or reaction time when compared to people without CAI (Koldenhoven et al., 2019a; Son et al., 2019).

Although the activity of the GM muscle in individuals with CAI has been investigated in prior studies (Fatima et al., 2020; Moisan et al., 2017), the relevant literature appears equivocal (Jaber et al., 2018; Levin et al., 2015; Mendes et al., 2021), and the role of GM activity in individuals with CAI warrants deeper evaluation (Labanca et al., 2021). Therefore, this study aimed to systematically review the literature to explore activity of the GM muscle in individuals with CAI.

## Methods

This systematic review was performed according to the PRISMA guidelines (Page et al., 2021), and has been registered at the international prospective register of systematic reviews (PROSPERO, registration number: CRD42022356875).

### Search Strategy

Studies were identified by searching electronic databases (PubMed, Embase, Cochrane Library, Web of Science, EBSCO and PEDro) from the year of inception to 31 October 2022, regardless of the language and the publication type. The terms and keywords used for searching articles were: (ankle instability OR instability, ankle OR unstable ankle) AND (gluteus medius* OR gluteus medius muscle* OR gluteus medium* OR gluteus medium muscle*) located within the title and/or abstract and/or keywords. Additionally, an updated search was conducted on the 10^th^ of June 2024, and no new studies met the inclusion criteria.

### Study Selection

The search results from each database were combined. After the removal of duplicates, the titles and abstracts were screened by two independent reviewers to identify the relevant studies that would undergo full-text review, based on the following inclusion criteria: (1) participant: individuals with CAI, (2) intervention: physical activities or functional movements such as walking, standing, and jumping, (3) comparator: healthy controls or copers who reported a history of ankle sprain but without the symptoms of CAI, (4) outcome: gluteus medius muscle activity, and (5) study design: a control study.

Trials conducted with animals, cadavers, simulators, or prostheses were excluded. In order to avoid interference from external factors, studies investigating footwear and auxiliary equipment/material were excluded, such as those involving foot orthoses, ankle braces/devices, and kinesiology taping. Finally, articles that compared barefoot with shod were also excluded because these studies aimed only to detect changes in EMG in activities while wearing shoes.

### Quality Assessment

The quality of the included studies was assessed using the Newcastle-Ottawa Quality scale (NOS) to evaluate the quality of case-control studies (Hartling et al., 2013; Oremus et al., 2012). This scale contains nine items that were marked with a star for each accomplished item: high quality (7–9 stars), moderate quality (4–6 stars), and poor quality (0–3 stars) (Lo et al., 2014; Stang, 2010). The NOS was applied by two independent reviewers, and any discrepancy among them was resolved by discussion with a third reviewer to reach consensus.

### Data Synthesis

The GM muscle activities were reported with EMG signals. However, due to the differences across studies in the type of EMG equipment used, the units of outcome measurement (such as amplitude, onset time, and response time), and the standards for setting variables (such as initial contact), the results could not be unified for direct comparison. Therefore, we analysed the difference in GM activity between participants with CAI and control groups. The analysis was presented as mean and standard deviation or a wave chart. When a *p*-value obtained was less than 0.05, statistical significance was concluded.

## Results

### Search Results

**Figure 1:**
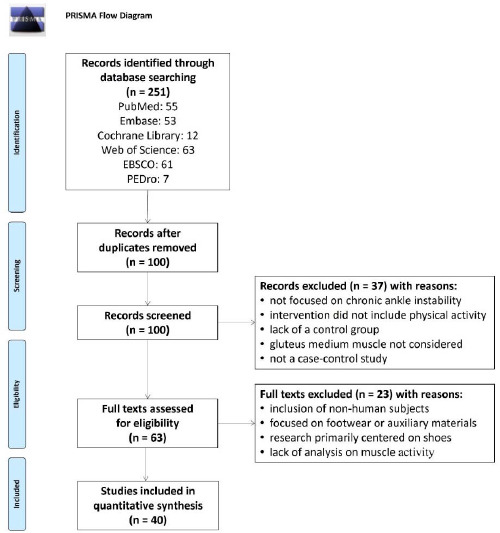
Flowchart of the study selection.

A total of 251 articles were retrieved from the initial literature search. After the removal of duplicates and papers that did not satisfy the inclusion criteria, 40 articles (1840 participants) were included in the review (Balasukumaran et al., 2020; Coglianese et al., 2011; Donahue et al., 2014; Drouin, 2002; Fatima et al., 2020; Feger et al., 2014; Feger and Hertel, 2015; Han et al., 2021; Jaber, 2017; Jaber et al., 2018; Jeong, 2021; Kazemi et al., 2017; Kim, 2015; Kim et al., 2019; Koldenhoven et al., 2016, 2019a, 2022; Koshino et al., 2016; Kunugi et al., 2018; Levin et al., 2012, 2015; Lin et al., 2021; Mendes et al., 2021; Moisan et al., 2020a, 2020b, 2021; Northeast et al., 2018; Rios et al., 2015; Son et al., 2017, 2019; Tajdini et al., 2022; Van Deun et al., 2007, 2011; Webster and Gribble, 2013; Webster et al., 2011, 2016; Wikstrom et al., 2010; Yalfani and Gandomi, 2016; Yousefi et al., 2019a, 2019b). The PRISMA flow diagram of the study selection process is shown in Figure 1.

### Quality of the Studies

The stars of the NOS for the included studies ranged from 5/9 to 9/9. A total of 37/40 studies were of high quality, and three out of 40 were of moderate quality (Webster et al., 2011; Yousefi et al., 2019a, 2019b). No study was found to be of low quality. Table 1 presents the NOS stars scored for each article.

**Table 1:** NOS stars of included studies.

Study	Selection				Comparability		Exposure/Outcome			Total stars
	S1	S2	S3	S4	C1	C2	E1	E2	E3	
Balasukumaran et al. (2020)	*	*	*	*	*	*	*	*	*	9
Coglianese et al. (2011)		*	*		*	*	*	*	*	7
Donahue et al. (2014)	*	*	*	*	*	*	*		*	8
Drouin (2002)	*	*	*		*		*	*	*	7
Fatima et al. (2020)	*	*	*		*	*	*	*	*	8
Feger et al. (2014)	*	*	*	*	*	*	*	*	*	9
Feger and Hertel (2015)	*	*	*	*	*	*	*	*	*	9
Han et al. (2021)	*	*	*		*	*	*	*	*	8
Jaber (2017)	*	*	*	*	*	*	*	*	*	9
Jaber et al. (2018)	*	*	*	*	*	*	*	*	*	9
Jeong (2021)	*	*	*		*	*	*	*	*	8
Kazemi et al. (2017)		*	*		*	*	*	*	*	7
Kim (2015)	*	*	*	*	*	*	*	*	*	9
Kim et al. (2019)	*	*	*	*	*	*	*	*	*	9
Koldenhoven et al. (2016)	*	*	*	*	*	*	*	*	*	9
Koldenhoven et al. (2019a)	*	*	*	*	*	*	*	*	*	9
Koldenhoven et al. (2022)	*	*	*	*	*	*	*	*	*	9
Koshino et al. (2016)	*	*	*	*	*	*	*	*	*	9
Kunugi et al. (2018)	*	*	*	*	*	*	*	*	*	9
Levin et al. (2012)	*		*	*	*	*	*	*	*	8
Levin et al. (2015)	*	*	*	*	*	*	*	*	*	9
Lin et al. (2021)	*	*	*	*	*	*	*	*	*	9
Mendes et al. (2021)	*	*	*	*	*	*	*	*	*	9
Moisan et al. (2020a)	*	*	*		*	*	*	*	*	8
Moisan et al. (2020b)	*	*	*	*	*	*	*	*	*	9
Moisan et al. (2021)	*	*	*	*	*	*	*	*	*	9
Northeast et al. (2018)	*	*	*	*	*	*	*	*	*	9
Rios et al. (2015)	*	*	*	*	*	*	*	*	*	9
Son et al. (2017)	*	*	*	*	*	*	*	*	*	9
Son et al. (2019)	*	*	*	*	*	*	*	*	*	9
Tajdini et al. (2022)	*	*	*	*	*	*	*	*	*	9
Van Deun et al. (2007)	*		*	*	*	*	*	*	*	8
Van Deun et al. (2011)	*	*	*	*	*	*	*	*	*	9
Webster et al. (2011)	*		*		*		*	*	*	6
Webster and Gribble (2013)	*	*	*	*	*	*	*	*	*	9
Webster et al. (2016)	*	*	*	*	*	*	*	*	*	9
Wikstrom et al. (2010)	*	*	*		*	*	*	*	*	8
Yalfani and Gandomi (2016)	*	*	*	*	*	*	*	*	*	9
Yousefi et al. (2019a)	*		*		*			*	*	5
Yousefi et al. (2019b)	*		*		*		*	*	*	6

NOS: Newcastle-Ottawa Scale; S1: Definition of cases; S2: Representativeness of the cases; S3: Selection of controls; S4: Adequate control definition; C1: Comparability of cases; C2: Study controls for the basis of the analysis; E1: Ascertainment of the exposure; E2: Ascertainment of the same method used for cases and controls; E3: Non-response rate

### Study Characteristics

Forty studies included in this review had a case-control design, and were published between 2002 and 2022. Additionally, all studies used EMG to investigate GM activity. Study and participant characteristics are presented in Table 2.

**Table 2a:** Characteristics of included studies—Part 1.

Study Task	Participants: Chronic ankle instability			Participants: Control			Electromyography instrumentation ** Test/placement
	§ Inclusion criteria	§§ Characteristics	†† Test limb	§ Inclusion criteria	§§ Characteristics	†† Test limb	
Balasukumaran et al. (2020) (1) Walking (2) Backward walking	# (i) (ii)	N=16, 8/8, 25.44 (2.39), 1.71 (0.11), 71.69 (13.82)	Unclear	(iv)	N=16, 9/7, 25.56 (3.44), 1.72 (0.10), 68.36 (12.44)	Unclear	Delsys, Boston, USA Φ
Coglianese et al. (2011) Jump-landing	* Unclear	N=10, 6/4, 23 (4), 1.80 (0.14), 80.9 (25)	Unclear	(iv)	N=10, 6/4, 23 (2), 1.81 (0.13), 81.6 (27)	Unclear	Unclear Unclear
Donahue et al. (2014) Perturbation	* (ii)	N=40 (total), 22 (3), 1.7 (0.13), 74.9 (16.1)	¦	(iv)	N=40 (total), 20 (2), 1.7 (0.11), 65.6 (12.9)	†	Biopac, Goleta, CA Ѳ
Drouin (2002) Functional exercises (stepping)	* (ii)	N=45, 19/26, 23.35 (6.59), 1.71 (0.09), 72.91 (11.71)	‡	(iv + v): 18+4	N=22, 13/9,24.77 (5.32), 1.70 (0.09), 68.85 (11.36)	†	Noraxon, Scottsdale, USA Ѳ
Fatima et al. (2020) (1) Balance (2) Functional exercises (single-leg squat with Swiss ball)	* (ii)	N=17, 11/6, 24.4 (2.03), 1.58 (0.08), 54.9 (8.75)	‡	(iv)	N=17, 11/6, 24.6 (2.57), 1.60 (0.07), 57.7 (8.93)	†	AD Instruments, Dunedin, NZ Φ
Feger et al. (2014) (1) Single-leg stance (2) Jump (3) Balance (4) Functional exercises (forward lunges)	# (i) (ii) (iii)	N=15, 10/5, 23 (4.2), 1.73 (0.11), 72.4 (14)	‡	(iv)	N=15, 10/5, 22.9 (3.4), 1.73 (0.09), 70.8 (18)	†	Bopac, Goleta, CA Ѳ
Feger and Hertel (2015) Walking	# (i) (ii) (iii)	N=15, 10/5, 23 (4.2), 1.73 (0.11), 72.4 (14)	‡	(iv)	N=15, 10/5, 22.9 (3.4), 1.73 (0.09), 70.8 (18)	†	Biopac, Goleta, CA Ѳ
Han et al. (2021) (1) Drop landing (2) Perturbation	* (ii) (iii)	N=20, 11/9, 23.5 (2.5), 1.70 (0.1), 70.6 (10.5)	Unclear	(iv)	N=20, 11/9, 23.4 (2.6), 1.73 (0.07), 70.5 (10.7)	Unclear	Unclear Unclear
Jaber (2017) Balance	# (i) (ii)	N=16, 9/7, 29.6 (4.2), 1.70 (0.06), 72.6 (16.9)	‡	(iv) (v)	N=16, 11/5, 25.8 (4.4), 1.71 (0.11), 73.9 (12.1) N=16, 5/11, 27.8 (4.4), 1.72 (0.07), 73.2 (9.6)	(iv): † (v): ‡	Noraxon, Scottsdale, USA Φ
Jaber et al. (2018) Balance	# (i) (ii)	N=16, 9/7, 29.6 (4.2), 1.70 (0.06), 72.6 (16.9)	‡	(iv) (v)	N=16, 11/5, 25.8 (4.4), 1.71 (0.11), 73.9 (12.1) N=16, 5/11, 27.8 (4.4), 1.72 (0.07), 73.2 (9.6)	(iv): † (v): ‡	Noraxon, Scottsdale, USA Φ
Jeong (2021) Drop landing	# (i) (ii)	N=21, 0/21, 24.19 (1.88), 1.80 (0.08), 81.33 (13.13)	‡	(iv)	N=9, 0/9, 24.44 (2.12), 1.80 (0.05), 85.33 (8.27)	†	Noraxon, Scottsdale, USA Ѳ
Kazemi et al. (2017) Perturbation	* (ii)	N=16, 16/0, 24.5 (3.75), 1.65 (0.06), 61.25 (6.49)	‡	(iv)	N=18, 18/0, 24.5 (3.32), 1.63 (0.06), 56.52 (8.18)	†	MIE Medical Research, UK Φ
Kim (2015) Jump	* (iii)	N=25, 23.3 (1.9), 1.77 (0.11), 70.9 (11.4)	‡	(iv)	N=25, 23.7 (2.5), 1.75 (0.11), 70.3 (12.8)	†	Delsys, Boston, USA Ѳ
Kim et al. (2019) (1) Jump-landing (2) Cutting	# (i) (ii) (iii)	N=100, 46/54, 22 (2.3), 1.74 (0.09), 72 (14)	‡	(iv)	N=100, 46/54, 22 (3.3), 1.73 (0.09), 71 (13)	†	Delsys, Boston, USA Ѳ
Koldenhoven et al. (2016) Walking	# (i) (ii) (iii)	N=17, 11/6, 20 (2.6), 1.70 (0.11), 77.4 (5.1)	‡	(iv)	N=17, 11/6, 21.8 (4.3), 1.67 (0.1), 75.9 (4.4)	†	Delsys, Boston, USA Ѳ
Koldenhoven et al. (2019a) Walking	# (i) (ii) (iii)	N=18, 16/2, 21.5 (3.4), 1.68 (0.09), 66.9 (14.4)	‡	(v)	N=18, 16/2, 20.5 (1.9), 1.68 (0.06), 66.2 (11.3)	‡	Delsys, Boston, USA Ѳ
Koldenhoven et al. (2022) Running	# (i) (ii) (iii)	N=13, 13/0, 20.7 (2.8), 1.67 (0.08), 66.5 (13.7)	‡	(v)	N=13, 13/0, 20 (0.8), 1.66 (0.05), 62.3 (8)	‡	Delsys, Boston, USA Ѳ
Koshino et al. (2016) (1) Walking (2) Cutting (3) Side-turn while walking	# (i) (ii)	N=10, 1/9, 21 (0.9), 1.74 (0.08), 65.9 (7.2)	‡	(iv)	N=10, 1/9, 20.8 (1.8), 1.74 (0.07), 66.5 (8.3)	†	Nihon Kohden, Tokyo, JPN Ѳ
Kunugi et al. (2018) Jump-landing	# (i) (ii)	N=15, 0/15, 19.8 (0.94), 1.73 (0.05), 66.59 (4.24)	‡	(iv)	N=15, 0/15, 20.07 (1.03), 1.74 (0.04), 68.31 (4.37)	least affected	Biometrics, Newport, UK Ѳ
Levin et al. (2012) Single-leg stance	# (i) (ii)	N=20, Matched control (age ± 1 year, BMI ± 5%)	¦	(iv)	N=20, 12/8, 21.8 (2.4), 1.64 (0.13), 68.4 (17.9)	†	Noraxon, Scottsdale, USA Ѳ
Levin et al. (2015) Jump-landing	# (i) (ii) (iii)	N=9, 5/4, 23.7 (4.24), 1.72 (0.09), 70.8 (13.2)	‡	(iv)	N=9, 4/5, 21.1 (1.36), 1.78 (0.1), 67.6 (10.7)	†	Mega Electronics, FIN Ѳ
Lin et al. (2021) (1) Walking (2) Perturbation	# (i) (ii)	N=13, 25.2 (4.5), 1.68 (0.08), 65.4 (9.5)	¦	(iv)	N=13, 26.8 (4), 1.69 (0.08), 63.3 (7.6)	†	Myon, CH Φ
Mendes et al. (2021) Jump-landing	# (i) (ii)	N=20, 0/20, 16.5 (1), 1.77 (0.1), 74.4 (13.6)	‡	(iv)	N=17, 0/17, 16.7 (1.4), 1.83 (0.09), 70.7 (16.1)	†	São José dos Campos, BRA Φ
**Please note that footnotes are provided after Table 2b**.							

**Table 2b:** Characteristics of included studies—Part 2.

Study Task	Participants: Chronic ankle instability			Participants: Control			Electromyography instrumentation ** Test/placement
	§ Inclusion criteria	§§ Characteristics	†† Test limb	§ Inclusion criteria	§§ Characteristics	†† Test limb	
Moisan et al. (2020a) Walking	# (i) (ii) (iii)	N=21, 17/4, 26.3 (8.5), 1.65 (0.08), 64.9 (12.7)	‡	(iv)	N=21, 17/4, 25.1 (5.3), 1.67 (0.09), 61.7 (12.7)	†	Delsys, Boston, USA Φ
Moisan et al. (2020b) Jump-landing	# (i) (ii) (iii)	N=32, 21/11, 25.3 (5.2), 1.68 (0.09), 72.3 (12.4)	Unclear	(iv)	N=31, 20/11, 23.7 (4), 1.7 (0.08), 67.1 (11.7)	Unclear	Delsys, Boston, USA Φ
Moisan et al. (2021) Walking	# (i) (ii) (iii)	N=28, 18/10, 25.5 (5.5), 1.69 (0.09), 71.3 (12.3)	‡	(iv)	N=26, 17/9, 23.7 (4.1), 1.7 (0.09), 67.3 (12.2)	†	Delsys, Boston, USA Φ
Northeast et al. (2018) Walking	# (i) (ii)	N=18, 5/13, 22 (2.7), 1.77 (0.08), 74.1 (9.6)	‡	(iv)	N=18, 4/14, 22.4 (3.6), 1.78 (0.08), 70.4 (11.9)	†	Biometrics, Gwent, UK Φ
Rios et al. (2015) Functional exercises (kicking a ball)	* (ii)	N=21, 13/8, 25 (20-31)	‡	(iv)	N=21, 13/8, 25 (21-31)	†	São José dos Pinhais, BRA Φ
Son et al. (2017) (1) Jump-landing (2) Cutting	# (i) (ii) (iii)	N=20, 8/12, 22.7 (2), 1.75 (0.1), 73.4 (12.1)	Unclear	(iv) (v)	N=20, 8/12, 21.8 (2.3), 1.73 (0.08), 69.2 (10.4) N=20, 8/12, 22.1 (2.1), 1.74 (0.08), 72.6 (12.3)	(iv): Unclear (v): Unclear	Delsys, Boston, USA Ѳ
Son et al. (2019) Walking	# (i) (ii) (iii)	N=100, 51/49, 22.2 (2.3), 1.74 (0.1), 70.8 (14.4)	‡	(iv)	N=100, 45/55, 22.5 (3.3), 1.73 (0.13), 72.6 (18.7)	†	Delsys, Boston, USA Ѳ
Tajdini et al. (2022) Walking	# (i) (ii) (iii)	N=28, 9/19, 23.2 (2.9), 1.74 (0.09), 68.5 (9.1)	‡ (right)	(iv)	N=28, 9/19, 24.3 (3.1), 1.75 (0.08), 70.9 (9.5)	† (right)	MIE Medical Research, UK Φ
Van Deun et al. (2007) Single-leg stance	* (i)	N=10, 4/6, 21.3 (4), 1.71 (0.07), 64.7 (6.8)	‡	(iv)	N=30, 17/13, 22.5 (2), 1.71 (0.06), 63 (7.5)	†	Noraxon, Scottsdale, USA Ѳ
Van Deun et al. (2011) Single-leg stance	# (i) (ii)	N=20, 12/8, 21.2 (2.1), 1.76 (0.1), 71.7 (11.3)	¦	(iv)	N=20, 12/8, 21.8 (2.4), 1.64 (0.13), 68.4 (17.9)	†	Noraxon, Scottsdale, USA Ѳ
Webster et al. (2011) Jump-landing	* Unclear	N=16, 8/8, 20.5 (2), 1.72 (0.11), 69.13 (13.31)	Unclear	(iv)	N=16, 8/8, 22 (3.3), 1.71 (0.1), 69.63 (14.82)	Unclear	Unclear Ѳ
Webster and Gribble (2013) Functional exercises (Rotational lunge and single leg rotational squat)	# (i) (ii) (iii)	N=9, 8/1, 20.9 (2.4), 1.65 (0.09), 68.1 (9.4)	‡	(iv)	N=9, 8/1, 22.9 (4.6), 1.65 (0.07), 65.4 (10)	†	Noraxon, Scottsdale, USA Ѳ
Webster et al. (2016) Jump-landing	# (i) (ii) (iii)	N=16, 8/8, 20.5 (2), 1.72 (0.11), 69.13 (13.31)	‡	(iv)	N=16, 8/8, 22 (3.3), 1.71 (0.1), 69.63 (14.82)	†	Noraxon, Scottsdale, USA Ѳ
Wikstrom et al. (2010) Walking	# (i) (ii)	N=20, 20.5 (1), 1.70 (0.1), 74.2 (20.2)	‡ plus uninjured	(iv)	N=20, 20.85 (1.6), 1.64 (0.08), 64.2 (10.62)	†	Konigsberg, Pasadena, CA Ѳ
Yalfani and Gandomi (2016) Sudden ankle supination	# (i) (ii) (iii)	N=25, 11/14, 19.5 (2.5), 1.71 (0.12), 70.6 (9.6)	‡	(iv)	N=25, 11/14, 19.6 (2.06), 1.70 (0.08), 63 (7.4)	†	Unclear Ѳ
Yousefi et al. (2019a) Walking	* Unclear	N=17, 22.3 (2.97), 1.76 (0.07), 66.8 (9.51)	Unclear	(iv)	N=17, 23 (1.68), 1.77 (0.06), 70.45 (6.93)	Unclear	Unclear Unclear
Yousefi et al. (2019b) Perturbation	* Unclear	N=7, 24.31 (0.81), 1.75 (0.04), 71.15 (7.21)	Unclear	(iv)	N=7, 23.4 (1.7), 1.76 (0.06), 72.25 (6.14)	Unclear	Myon, CH Unclear

§ The inclusion criteria were as follows: (i) A history of at least 1 significant ankle sprain (for reference, the initial sprain must have occurred at least 12 months prior to study enrollment, was associated with inflammatory symptoms (pain, swelling, etc.), created at least 1 interrupted day of desired physical activity, and the most recent injury must have occurred more than 3 months prior to study enrollment.); (ii) a history of previously injured ankle joint “giving way” and/or recurrent sprain and/or “feelings of instability”; for the self-reported ankle instability, it should be confirmed with a validated ankle instability specific questionnaire such as the Ankle Instability Instrument (AII), the Cumberland Ankle Instability Tool (CAIT), and the Identification of Functional Instability scale (IdFAI), using the associated cut-off score; (iii) a general self-reported foot and ankle function questionnaire (e.g., Foot and Ankle Ability Measure (FAAM) and the Foot and Ankle Outcome Score (FAOS)) that is used to describe the level of disability of the cohort, but this is not required for the inclusion criteria of chronic ankle instability; (iv) Healthy controls, participants with no history of ankle sprain; (v) Copers, participants reported a history of unilateral ankle sprain but without complaint of disability and/or “giving way” episodes since the injury. The symbol (#) means that inclusion criteria for the chronic ankle instability were relatively in accordance with the International Ankle Consortium recommendations. The symbol (*) means that the ankle instability was involved in inclusion criteria, but it failed to explicitly meet minimum standard inclusion criteria endorsed by the International Ankle Consortium for enrolling individuals with chronic ankle instability, or the subjects were only defined as functional ankle instability rather than chronic ankle instability, or there was no clear explanation. §§ Data were presented in the following order: number, sex (F/M), age (years), height (m), weight (kg). †† The test limbs were as follows: ‡ Chronic ankle instability (injured limb) involved. ¦ Chronic ankle instability involved and no bilateral chronic ankle instability. ‡ Chronic ankle instability (injured limb) involved or the worse limb in the case of bilateral chronic ankle instability (sprains). † Unilateral dominant limb; the dominant limb was reported in the original text, which was defined as the leg used to kick a ball. † Unilateral limb regardless of dominance. † Matched (right or left) to involved chronic ankle instability or a side-matched control to chronic ankle instability (limb dominance was not controlled during matching) or matched with respect to age, body mass index, and lower limb dominance. ** There were two main types of tests as follows: Φ Electrode placement was performed according to Surface Electromyography for the Non-Invasive Assessment of Muscles (SENIAM) guidelines. Ѳ An introduction of the electrode placement was made, but it was not defined whether it was in line with the SENIAM guidelines.

Five notable aspects of the included studies were as follows: (1) task: a total of seven tasks were involved in the included studies (walking (14 studies) (Balasukumaran et al., 2020; Feger and Hertel, 2015; Koldenhoven et al., 2016, 2019a, 2022; Koshino et al., 2016; Lin et al., 2021; Moisan et al., 2020a, 2021; Northeast et al., 2018; Son et al., 2019; Tajdini et al., 2022; Wikstrom et al., 2010; Yousefi et al., 2019a), stance-transition (four studies) (Feger et al., 2014; Levin et al., 2012; Van Deun et al., 2007, 2011), jump-landing (13 studies) (Coglianese et al., 2011; Feger et al., 2014; Han et al., 2021; Jeong, 2021; Kim, 2015; Kim et al., 2019; Kunugi et al., 2018; Levin et al., 2015; Mendes et al., 2021; Moisan et al., 2020b; Son et al., 2017; Webster et al., 2011, 2016), perturbation (six studies) (Donahue et al., 2014; Han et al., 2021; Kazemi et al., 2017; Lin et al., 2021; Yalfani and Gandomi, 2016; Yousefi et al., 2019b), balance (four studies) (Fatima et al., 2020; Feger et al., 2014; Jaber, 2017; Jaber et al., 2018), cutting (three studies) (Kim et al., 2019; Koshino et al., 2016; Son et al., 2017), and other functional exercises (seven studies) (Balasukumaran et al., 2020; Drouin, 2002; Fatima et al., 2020; Feger et al., 2014; Koshino et al., 2016; Rios et al., 2015; Webster and Gribble, 2013)); (2) CAI inclusion criteria: there were 12 studies that were not in accordance with the International Ankle Consortium recommendations (Coglianese et al., 2011; Donahue et al., 2014; Drouin, 2002; Fatima et al., 2020; Han et al., 2021; Kazemi et al., 2017; Kim, 2015; Rios et al., 2015; Van Deun et al., 2007; Webster et al., 2011; Yousefi et al., 2019a, 2019b); (3) test limb: although the test limbs in the CAI group were mostly on the affected side, the test limbs in the control group varied considerably (dominant limb: 11 studies (Donahue et al., 2014; Fatima et al., 2020; Jaber, 2017; Jaber et al., 2018; Koshino et al., 2016; Lin et al., 2021; Mendes et al., 2021; Rios et al., 2015; Tajdini et al., 2022; Van Deun et al., 2007; Webster and Gribble, 2013), matched to involved CAI: 7 studies (Feger et al., 2014; Feger and Hertel, 2015; Kazemi et al., 2017; Kim, 2015; Kim et al., 2019; Webster et al., 2016; Yalfani and Gandomi, 2016), random: 11 studies (Drouin, 2002; Jeong, 2021; Koldenhoven et al., 2016; Levin et al., 2012, 2015; Moisan et al., 2020a, 2021; Northeast et al., 2018; Son et al., 2019; Van Deun et al., 2011; Wikstrom et al., 2010), least affected: 1 study (Kunugi et al., 2018), injured limb in copers: 5 studies (Jaber, 2017; Jaber et al., 2018; Koldenhoven et al., 2019a, 2022; Son et al., 2017), no information: 8 studies (Balasukumaran et al., 2020; Coglianese et al., 2011; Han et al., 2021; Moisan et al., 2020b; Son et al., 2017; Webster et al., 2011; Yousefi et al., 2019a, 2019b)); (4) control group: most of the participants in the control groups were healthy, while there were 6 studies where the controls included copers (Drouin, 2002; Jaber, 2017; Jaber et al., 2018; Koldenhoven et al., 2019a, 2022; Son et al., 2017); (5) EMG measurement: not only were the devices different, but the procedures for testing also varied from study to study (there were 13 studies that were performed according to Surface Electromyography for the Non-Invasive Assessment of Muscles (SENIAM) guidelines) (Balasukumaran et al., 2020; Fatima et al., 2020; Jaber, 2017; Jaber et al., 2018; Kazemi et al., 2017; Lin et al., 2021; Mendes et al., 2021; Moisan et al., 2020a, 2020b, 2021; Northeast et al., 2018; Rios et al., 2015; Tajdini et al., 2022).

### Gluteus Medius Muscle Activity

The data were organized according to seven tasks. However, there was substantial variation in the EMG muscle activity outcome measurements, such as maximal voluntary isometric contraction (MVIC) amplitude, the area under the root mean square (RMS), onset time, and response time. Also, the data acquisition and processing protocols varied, especially in the identification of the periods for pre- and post- initial contact (IC). Given these differences and the five aspects mentioned above, when comparing individuals with CAI to healthy controls, the results reported by various studies for each task were inconsistent. Gluteus medius activity during various tasks is presented in Table 3.

**Table 3a:** Gluteus medius muscle activation during various tasks—Part 1.

Walking/Running		
Study	Main outcome measurement	Summary results of individuals with CAI versus the control group
Balasukumaran et al. (2020) (1)	MVIC AUC at pre-IC and post-IC	EMG amplitude activity across the gait cycle: ※ (▽) The pre-IC and post-IC AUC values: ※ (▽)
Feger and Hertel (2015)	RMS Area under the RMS curve	Time of Activation Relative to Initial Contact: ※ (▲) Percentage of Activation Time per stride cycle: ※ (▲) Pre and Post IC Walking Amplitude: ※ (▲)
Koldenhoven et al. (2016)	Area under the EMG RMS curve and EMG amplitudes	Area under the EMG RMS curve for 100 ms pre-IC: ▽ Area under the EMG RMS curve for 200 ms post-IC: ※ (▲) EMG amplitudes during the final 50 % of the stance and first 25 % of swings: ▽
Koldenhoven et al. (2019a)	RMS Amplitude (%)	No group differences were identified for EMG variables; however, the coper group trended towards higher GM RMS amplitude compared to the CAI group during the stance phase for all walking speeds. ※ (▽)
Koldenhoven et al. (2022)	RMS Amplitude (%)	There were no significant differences identified for GM sEMG amplitude throughout the gait cycle: ※ (▲)
Koshino et al. (2016) (1)	MVIC	There were no significant group differences in the mean EMG activities for GM: ※ (▲)
Lin et al. (2021) (1)	RMS Onset times	The magnitude of muscle activation: at Pre200 and Post100 ms, GM of both sides was activated less in the CAI group than in controls. ※ (▽) The onset of muscle activation: after heel contact, the CAI group activated GM of both sides earlier than controls. ※ (▲)
Moisan et al. (2020a)	RMS	Comfortable walking: the CAI group exhibited a decreased GM activity from 6 to 9% and 99 to 100% of the stance phase compared to the control group. ○ Fast walking: ※ (▽)
Moisan et al. (2021)	RMS	No between-group differences in GM activity were found. ※ (▽)
Northeast et al. (2018)	MVIC	No significant differences were observed in the GM activation in either phase of the gait between the matched control and the CAI group’s affected limb. ※
Son et al. (2019)	Amplitude, % of reference	4% less GM EMG activity throughout most of the stance ○
Tajdini et al. (2022)	MVC	The asymmetry of GM during the contact phase, ※ (▲) The asymmetry of GM during the mid-stance/propulsion phase, ▽ Significantly greater GM activity for the injured limb of individuals with CAI compared to the dominant limb of the control group. ▽
Wikstrom et al. (2010)	Amplitude, %	Planned gait termination EMG: GM activation increased during phase no. 4 (second peak loading to toe off). For the swing limb, it was activated to a greater degree than during a comparable phase in normal walking. Group differences were evident for GM on the lead limb. Subjects with CAI activated the GM to a greater extent than the control group. No such main effects were observed for the swing limb. ▽ Unplanned gait termination EMG: similar to planned stopping, the expected phase effects were noted with increases in GM activity during a late stance (phase 4). Lead limb activity of the GM was greater than the equivalent phases of the normal gait. ▽
Yousefi et al. (2019a)	Muscle activation	There was no significant difference in terms of muscle activity of the GM between groups. ※
**Stance/Transition**		
**Study**	**Main outcome measurement**	**Summary results of CAI versus Control**
Feger et al. (2014) (1)	Area under the RMS curve MVIC	Isolated Muscle Activation: ※ (▲)
Levin et al. (2012)	Onset times	Subjects with CAI showed significantly slowing in onset times of GM as compared with healthy controls. ○
Van Deun et al. (2007)	Onset times	Onset of muscle activity, in both of EO and EC conditions, GM showed a significantly later onset in subjects with CAI compared with control subjects. ○
Van Deun et al. (2011)	Onset times	No between-group differences were found in the onset times of GM activity. ※ (▽) CAI group were generally later than control group in the onset times of GM activity.
**Jump/Landing**		
**Study**	**Main outcome measurement**	**Summary results of individuals with CAI versus the control group**
Coglianese et al. (2011)	Onset time (ms) EMG amplitude	Mean GM pre-activation occurred 57 ms earlier for the controls: ▽ Mean EMG amplitudes during landing: ※ (▲)
Feger et al. (2014) (2)	Pre-IC and Post-IC area under the RMS curve	Pre-IC: ※ (▽) Post-IC: ※ (▽)
Han et al. (2021) (1)	RMS	No group differences were identified for GM activation during a drop landing: ※
Jeong (2021)	MVIC	GM activity at the IC and the point of peak knee flexion: ※ (▲)
Kim (2015) (1)	EMG Amplitudes. (% of Reference. Value)	The CAI group demonstrated decreased GM EMG amplitude during 0–5%, 40–42% and 90–100% of the stance phase, while the control group decreased EMG amplitude of the GM between 82 and 100% of the stance phase. A significant difference was observed during 30–35% and 80–85% of the stance phase. ▽
Kim et al. (2019)	EMG Amplitudes. (% of Reference. Value)	Relative to controls, individuals with CAI displayed up to 5.2% greater GM activity during 32%–40% of ground contact (*p* < 0.05). ▽
Kunugi et al. (2018)	MVC	No group differences were identified for GM activity: ※ (▽)
Levin et al. (2015)	RMS (normalized)	No = no inversion, 50% = chance for inversion at 50% of the jumps, 100% = inversion at all jumps. Pre-impact period: non-inverting and inverting side, ※ (▲). Post-impact period: short latency reflex responses: no period, ○; Long latency reflex responses: all period, ※
Mendes et al. (2021)	Magnitude Onset times	Integrated linear envelope: Landing, ※ (▲); Propulsion, ※ (▲) Onset: Landing, ※ (▽); Propulsion, ▽. In the propulsion phase, the CAI group showed a significant lower muscle activation onset with a strong effect size when compared to the control group in regard to the GM
Moisan et al. (2020b)	RMS	For the side jump landing task: GM activity decreased from 0 to 5% of the pre-activation phase for the CAI group. ○ For the drop landing task: No between-group difference was observed on the even surface. ※ No between-group difference was observed on the unstable surface. ※ The CAI group exhibited decreased muscle activity of the GM from 87 to 100% of the pre-activation phase in the laterally inclined surface. ○
Son et al. (2017) (1)	EMG Amplitudes. (% of Reference. Value)	Relative to controls, CAI individuals displayed up to 13% greater GM activity during 3% to 14% of the stance, ▽ and 8% less GM activity during 35% to 45% of the stance. ○ Relative to copers, CAI individuals displayed up to 16% less GM activity during 0% to 2% and 35% to 74% of the stance. ○

**Table 3b:** Gluteus medius muscle activation during various tasks—Part 2.

Walking/Running		
Study	Main outcome measurement	Summary results of individuals with CAI versus the control group
Webster et al. (2011)	% Peak muscle activation	There were no statistical differences between groups for the GM as well as no influence of fatigue. ※ (▽)
Webster et al. (2016)	EMG Amplitudes	GM activity pre-landing results by group, ※ (▽) GM activity pre-landing in pre-fatigue and post-fatigue, ※ (▽)
**Perturbation/Sudden event**		
**Study**	**Main outcome measurement**	**Summary results of individuals with CAI versus the control group**
Donahue et al. (2014)	Onset time (ms) Z-scores	The latencies were not significantly different in the non-perturbed side between the FAI and control groups: ※; and the Short-latency reflex onsets were not significantly different between the two groups in the GM of the perturbed side: ※. The short-latency reflex amplitude, determined by the average EMG from a period of the reflex onset latency to 40 ms after the reflex onset, was z-scored to compare across groups. There were no significant differences in the short-latency reflex z-scores for the GM of the perturbed side: ※. Long-latency reflex amplitudes were based on a period of EMG activity 40 ms after the onset of the short-latency reflex-up to 80 ms after the short-latency reflex. Again, to normalize across conditions the long-latency amplitudes were then transformed to z-scores. There were no other significant differences in the long-latency reflex z-scores for the GM of the perturbed side: ※.
Han et al. (2021) (2)	RMS	No group differences were identified for GM activity after ankle perturbation: ※
Kazemi et al. (2017)	Onset time MVC	Onset time, no significant difference in GM between the two groups: ※ (▲) Amplitude, no significant difference in GM between the two groups: ※ (▽)
Lin et al. (2021) (2)	RMS Onset times	The magnitude of muscle activation: at Pert200, GM of both sides activated less in the CAI group than control. ※ (▽) The onset of muscle activity: after perturbation, no significant difference in GM between the two groups. ※ (▲)
Yalfani and Gandomi (2016)	MVC	Feed-forward EMG was different between healthy controls and the CAI group. ▽ The feed-forward EMG in the CAI group was significantly higher than that in the control group. Feed-back EMG was no significantly different between healthy controls and the CAI group. ※ (▽)
Yousefi et al. (2019 (b))	Response time	The response time to perturbation in GM was significantly lesser in subjects with functional ankle instability. ○
**Balance (SEBT/Y test)**		
**Study**	**Main outcome measurement**	**Summary results of individuals with CAI versus the control group**
Fatima et al. (2020) (1)	MVIC	EMG activity of the GM muscle was significantly different between individuals with CAI and healthy controls in three directions: ○
Feger et al. (2014) (3)	Area under the RMS curve MVIC	No significant differences were identified between the groups during SEBT trials in any direction: ※ (▲)
Jaber (2017)	MVIC MVC	No significant differences were identified between the CAI and healthy groups during SEBT trials in any direction: ※ (Anterior, Posteromedial: ▲; Medial, Posterolateral: ▽) No significant differences were identified between the CAI and coper groups during SEBT trials in any direction: ※ (Anterior, Medial, Posteromedial: ▲; Posterolateral: ▽)
Jaber et al. (2018)	MVIC Onset time	EMG activity amplitudes: there were no significant differences identified between the CAI and healthy groups for the three directions: ※ (Anterior, Posteromedial: ▲; Posterolateral: ▽) There were no significant differences identified between the CAI and coper groups for the three directions: ※ (Anterior, Posteromedial: ▲; Posterolateral: ▽) Muscle activity onset time: There was a significant difference in mean GM muscle activity onset time (seconds) among individuals with CAI, copers and controls in the posteromedial direction (1.4 ± 0.3 vs. 0.8 ± 0.2 vs. 0.9 ± 0.2, *p* = 0.038). The difference was statistically significant between CAI and coper groups (*p* = 0.035). ○
**Cutting/Side-cut**		
**Study**	**Main outcome measurement**	**Summary results of individuals with CAI versus the control group**
Kim et al. (2019) (2)	EMG Amplitudes. (%)	No group differences were found during 50%–100% of ground contact. ※
Koshino et al. (2016) (2)	%MVIC	There were no significant group differences in the mean EMG activities for GM: ※ (▽)
Son et al. (2017) (2)	EMG Amplitudes. (%)	No group differences were found during 51%–100% of the stance between individuals with CAI and controls/copers. ※ (▽)
**Other functional exercises**		
**Study**	**Main outcome measurement**	**Summary results of individuals with CAI versus the control group**
Balasukumaran et al. (2020) (2)	MVIC AUC at pre-IC and post-IC	EMG amplitude activity across the gait cycle: ※ (▽) The pre-IC and post-IC AUC values: ※ (▽)
Drouin (2002)	Onset times MVIC	Muscular onset and amplitude during stepping activities: ※ (▲)
Fatima et al. (2020) (2)	MVIC	EMG activity of the GM muscle was significantly different between individuals with CAI and healthy controls in a single-leg squat with and without a Swiss ball: ○
Feger et al. (2014) (4)	Pre-IC and Post-IC area under the RMS curve	Pre-IC: ※ (▽) Post-IC: ※ (▽)
Koshino et al. (2016) (3)	%MVIC	There were no significant group differences in the mean EMG activity for the GM: ※ (▽)
Rios et al. (2015)	EMG integral sum	For the GM, participants with CAI exhibited higher EMG during the simultaneous and compensatory postural adjustments as compared to the controls. ※ (▲)
Webster and Gribble (2013)	MVC	No group differences were found in the rotational lunge. ※ (▲) No group differences were found in the rotational squat. ※ (▽)

CAI: chronic ankle instability; GM: Gluteus medius; EMG: electromyography; sEMG: surface electromyography; RMS: root mean square; IC: initial contact; AUC: area under the curve; MVIC: maximal voluntary isometric contraction; MVC: maximum voluntary contraction; GRF: ground reaction force; COP: center of pressure; EO: eyes open; EC: eyes closed; USI: musculoskeletal ultrasound imaging; SEBT: star excursion balance test; YBT: Y-Balance Test; LAS: lateral ankle sprain. ※: No significant differences between groups (p > 0.05); ▲: Higher/earlier overall mean in the CAI group; ▽: Higher/earlier overall mean in the control group; ▽: Significantly favors the CAI group (p < 0.05); ○: Significantly favors the control group (p < 0.05).

In order to focus on the individuals with CAI, studies that did not meet the CAI inclusion criteria (International Ankle Consortium recommendations) were further excluded (Delahunt et al., 2010; Gribble et al., 2014; Martin et al., 2021). However, the results of the included studies were also not consistent, and a summary is presented in Table 4.

**Table 4:** Summary results from studies of gluteus medius muscle activation focusing on CAI.

Task	No significant difference (individuals with CAI versus healthy controls)	Significant differences reported in certain cases ‡			
		Favor individuals with CAI	Favor healthy controls	Favor both individuals with CAI and healthy controls	Favor copers
Walking/Running: 13 studies	8 studies	3 studies	2 studies		
Stance/Transition: 3 studies	1 study		2 studies		
Jump/Landing: 9 studies	4 studies	2 studies	2 studies	1 study	1 study
Perturbation/Sudden event: 2 studies	1 study	1 study			
Balance (SEBT/Y test): 3 studies	3 studies				1 study
Cutting/Side-cut: 3 studies	3 studies				
Other functional exercises: 4 studies	4 studies				

‡ : “Favor” means the participants’ gluteus medius muscle were more active

## Discussion

To the best of our knowledge, this is the first study to systematically review GM muscle activity in individuals with CAI. Previous muscle studies with CAI have focused on calf and foot musculature (Han et al., 2022), with relatively few investigations on the GM muscle. More studies are needed to confirm the effect of the GM in CAI, because it plays an important role in the stability of the lower limb during physical activities (Labanca et al., 2021).

Given its role in walking or running gait control, dysfunction of the GM muscle has been clinically implicated in the pathomechanics of CAI (DeJong et al., 2020a; So et al., 2022). Due to its anatomical attachment to the ilium, it has been reported that weak activity of the GM may have a negative effect on the control of pelvic and hip movements (DeJong et al., 2022; Labanca et al., 2021). In addition, an increasing number of studies have suggested that GM activity also has an impact on ankle stability (Northeast et al., 2018; Han et al., 2021). Specifically, the GM muscle attaches to the greater trochanter of the femur and iliac crest (DeJong et al., 2019); during a single-leg standing or landing task, the femur is immobile (the supporting leg is relatively fixed), and the contraction of GM muscle mainly acts on the ilium to stabilize the pelvis (DeJong et al., 2019, 2020b). If the GM muscle is unable to generate enough force to maintain the pelvic position during a contralateral leg swing, this would lead to an increase in the lower limb landing angle (deviation from the vertical line) (Besomi et al., 2020), which may lead to unwanted ankle inversion and a higher risk of ankle injury (Martin et al., 2021; McMullen et al., 2011).

From this point of view, further clarifying the effect of the GM muscle in CAI would be of great significance, and could support the design of rehabilitation programs for the management of CAI. However, many issues remain unresolved in the current studies on the GM muscle activity in individuals with CAI.

Firstly, the studies analysed in this review do not provide unequivocal evidence for or against the hypothesis that GM activity is affected in CAI. Due to the contradictory findings of the current research, the characteristics of GM muscular activity in CAI could not be analyzed using meta-analysis, and the extent to which individuals with CAI and healthy controls differ is also not clear. It is possible that differences in the participants involved in different studies contribute to this discrepancy. However, even if the studies that did not meet the inclusion criteria for CAI (International Ankle Consortium) were excluded (Delahunt et al., 2010; Gribble et al., 2014), there were still inconsistent results in certain tasks.

Overall, GM muscle activity during more functional tasks, such as balance, cutting, and complex exercises was poorly investigated. Thus, future studies should include these functional tasks to provide comprehensive data for better understanding of GM muscle activity characteristics in individuals with CAI, so that effective management programs can be developed.

In addition, regarding the quality of the studies, the NOS scores of included studies were generally high, and they met the NOS requirements for a case-control study (Lo et al., 2014; Stang, 2010). However, results were diverse, possibly due to different methods. Therefore, for the investigation of GM in individuals with CAI, there is a need for standardization of methodologies so as to improve comparability between studies and promote the evidence level of summary results.

In summary, considering the available research data on GM activity in CAI, reported EMG results have not shown a consistent pattern. This may be due to differences in the study design and outcome measures.

In terms of the study design, there were five issues evident in the studies included.

(1) The experimental tasks were diverse. There were simple tasks such as walking and landing, and there were also challenging movements such as cutting and dynamic balance, even though these tasks were insufficiently investigated since they were included in only few studies. Also, for certain tasks involving multifunctional exercises, these were designed with movements that are not standardized tasks commonly used in the management of CAI (Delahunt et al., 2018; Martin et al., 2021), such as a rotational lunge, a squat with rotational reach, and a single-legged squat with a Swiss ball (Fatima et al., 2020; Feger et al., 2014; Webster and Gribble, 2013); and thus these cannot be compared with other common tasks such as walking and standing. Therefore, it is suggested that researchers standardize test measures so that it would be possible to compare them more directly and clearly (Norris and Trudelle-Jackson, 2011; Stanek et al., 2011; Ye-ji et al., 2017). Moreover, in training and rehabilitation of individuals with CAI, it may not be necessary to get them to perform overly unique tasks (Han et al., 2022; Shi et al., 2019; Tan et al., 2022), since regular exercise therapies have been well established and proven to be effective in the management of CAI (Luan et al., 2021; Martin et al., 2021).

(2) The inclusion criteria for determining CAI in various studies were inconsistent, and not all studies referred to the CAI selection standards in the International Ankle Consortium recommendations (Delahunt et al., 2018; Gribble et al., 2014). This is a common issue in current CAI studies (Gribble et al., 2014). It is suggested that the inclusion criteria for CAI should meet at least three major conditions: history of ankle sprain, feelings of instability or giving way, and self-reported foot and ankle function (Delahunt et al., 2010; Hertel and Corbett, 2019).

(3) The choice of the test limb was not consistent. Although the affected side was mainly tested in the CAI group, the test limb of the control group varied from study to study. Some employed the dominant limb, some matched the affected side of CAI participants, and some set the test side without any requirements, which caused heterogeneity in the integrated analysis (Han et al., 2016; Wikstrom et al., 2010; Ye-ji et al., 2017). Accordingly, future studies should focus on the side contralateral to the CAI limb involved (unaffected side or a better limb in the case of bilateral CAI), because it is also related to hip stability as well as to postural control in individuals with CAI (Han et al., 2013, 2021; Steinberg et al., 2017).

(4) The characteristics of participants in the control groups were different. Specifically, healthy controls were different from copers who had experienced one significant ankle sprain although they were without any residual disability (Jaber, 2017; Jaber et al., 2018; Koldenhoven et al., 2019b), and the extent of this difference needs to be further clarified (Son et al., 2017). Based on certain similarities between CAI individuals and copers, such as their history of ankle sprain, they should be compared independently and discussed separately from the results of CAI versus healthy control comparisons.

(5) The protocols for EMG testing, especially in terms of the electrode placement, varied from study to study, and only few trials were conducted referring to the Surface Electromyography for the Non-Invasive Assessment of Muscles (SENIAM) guidelines (McCrary et al., 2018; Oliveira Ade and Goncalves, 2009). It is recommended that the processes implemented for EMG follow a uniform standard, as this could increase the reliability and comparability of the results from different studies.

With regard to outcome measures, two factors seriously hindered the synthesis of results. One was the selection of EMG outcomes. Some studies used onset time or response time, and some trials adopted EMG amplitude. Further, various units of amplitude were employed, such as the percentage of reference value and the area under the root mean square curve (Fatima et al., 2020; Moisan et al., 2020a; Oliveira Ade and Goncalves, 2009). The other area of variation was the acquisition of EMG data. There were substantial differences in the division of the period of pre- and post-initial foot contact, the definition of the landing phase (heel-strike, toe-strike, heel-off, toe-off), and the identification of the stage of the take-off (pre-activity, propulsion, swing, pre-landing).

Admittedly, EMG, as a tool for gauging GM muscle activity, is sensitive to variations in electrode placement, skin impedance, and signal processing techniques; this inherent susceptibility may compromise the reliability of results, especially when comparing muscle activity across different tasks (Claiborne et al., 2009; Green et al., 2019; Feger and Hertel, 2015). Suboptimal reliability in these aspects may lead to inconsistencies in muscle activity measurements, potentially contributing to the divergent outcomes observed in various studies. Further, these inherent challenges underscore the need for meticulous attention to methodological consistency and standardization in EMG assessments, ensuring that the reported outcomes accurately reflect the underlying physiological phenomena.

Finally, after the studies that did not meet the selection criteria for individuals with CAI recommended by International Ankle Consortium were excluded (Gribble et al., 2014), from the complexity of tasks, there was an interesting phenomenon. That was, for single modes of movement, such as walking, standing, and landing, the preference of GM activity fluctuated between individuals with CAI and healthy controls, whereas for complex modes of movement, such as cutting, multi-functional exercise, and complex dynamic balance control tasks, there was no significant difference in GM activity between these two groups. While the number of studies involved here was relatively small, this aspect raises future research possibilities.

## Study Limitations

One of the limitations of this review is the small sample size in some tasks in the investigation of muscle activity, thus the evidence here may be insufficient. In addition, the methodology of the included studies was not homogeneous, and there were also some objective differences between studies, such as the equipment type, the implementation environment, and the experimental process, which may also result in the inconsistent findings (McCrary et al., 2018). Finally, although all participants included had CAI, there was high heterogeneity between studies on the basis of their demographics, especially in profession and age, and gender differences were poorly investigated.

## Conclusions

There is currently no consensus regarding differences in GM activity between individuals with and without CAI, and the methodological variations in the studies with GM activity measurements decrease the possibility of generalising patterns. The findings suggest that a consensus on a standardized research protocol should be developed urgently to guide future studies on GM muscle activity in individuals with CAI.
